# Supplementary material for: Transcriptomic Insights into Mechanisms of Early Seed Maturation in the Garden Pea (Pisum sativum L.)
Source: Cells. 2020 Mar 23;9(3):779. doi: 10.3390/cells9030779 (PMC7140803; doi:10.3390/cells9030779)

Sprint−2(10) vs Sprint−

Global Summary

%DE = 0.84

genes with fdr < 0.2 = 1693 (867 + / 826 −)

genes with fdr < 0.1 = 1536 (805 + / 731 −)

genes with fdr < 0.05 = 1419 (751 + / 668 −)

genes with fdr < 0.01 = 1023 (581 + / 442 −)

genes in genesets = 0

<FC> = 0

<t−score> = 0.71

<p−value> = 0.01

<fdr> = 0.16

Global Genelist

Rank	ID	log(FC)	p−value / fdr / Metagene	Description
1	TRINITY_DN1	−0.92	0.000000010000014 x 30	
2	TRINITY_DN1	−0.68	0.000000020000014 x 30	
3	TRINITY_DN1	−0.43	0.000000020000012 x 27	
4	TRINITY_DN7	0.33	0.0000000200000124 x 1	
5	TRINITY_DN5	0.48	0.0000000300000125 x 1	
6	TRINITY_DN8	0.62	0.0000000300000530 x 4	
7	TRINITY_DN7	0.29	0.0000000600000530 x 8	
8	TRINITY_DN4	−17.38	0.000000070000051 x 30	
9	TRINITY_DN2	−0.72	0.000000090000055 x 30	
10	TRINITY_DN3	−0.27	0.000000090000056 x 5	
11	TRINITY_DN1	0.4	0.0000000100000730 x 6	
12	TRINITY_DN1	0.85	0.0000000130000730 x 3	
13	TRINITY_DN5	0.48	0.0000000140000725 x 1	
14	TRINITY_DN8	1.53	0.0000000190000730 x 1	
15	TRINITY_DN5	1.09	0.0000000200000728 x 1	
16	TRINITY_DN9	0.12	0.0000000270000723 x 18	
17	TRINITY_DN5	−0.5	0.0000000290000725 x 30	
18	TRINITY_DN7	0	0.0000000290000717 x 14	
19	TRINITY_DN8	0.56	0.0000000300000726 x 1	
20	TRINITY_DN3	0.46	0.0000000300000730 x 6	

Portrait

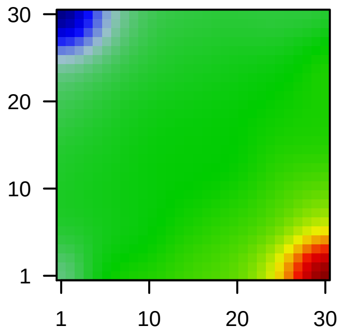

Top 100 DE genes

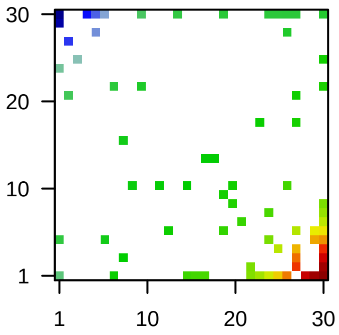

p−values

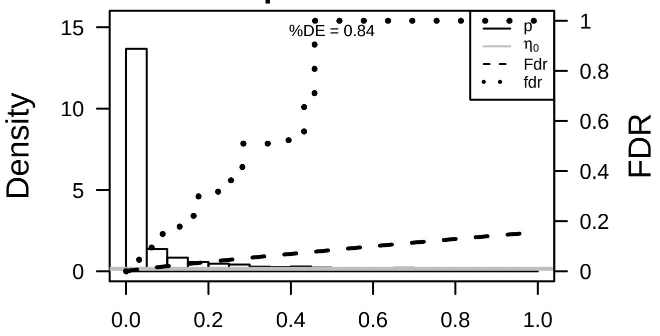

Supplement: Supplementary file 1 [file cells-09-00779-s001.zip › Supplementary materials/FileS4.zip!/FileS3/TFs - Results/Summary Sheets - Differences/Reports/Sprint.2.10..vs.Sprint.2.20..pdf]
